# The role of prefrontal–subcortical circuitry in negative bias in anxiety: Translational, developmental and treatment perspectives

**DOI:** 10.1177/2398212818774223

**Published:** 2018-05-08

**Authors:** Christina O. Carlisi, Oliver J. Robinson

**Affiliations:** 1 Division of Psychology and Language Sciences, University College London, London, UK; 2 Institute of Cognitive Neuroscience, University College London, London, UK

**Keywords:** Anxiety, circuit, negative bias, prefrontal cortex

## Abstract

Anxiety disorders are the most common cause of mental ill health in the developed world, but our understanding of symptoms and treatments is not presently grounded in knowledge of the underlying neurobiological mechanisms. In this review, we discuss accumulating work that points to a role for prefrontal–subcortical brain circuitry in driving a core psychological symptom of anxiety disorders – negative affective bias. Specifically, we point to converging work across humans and animal models, suggesting a reciprocal relationship between dorsal and ventral prefrontal–amygdala circuits in promoting and inhibiting negative bias, respectively. We discuss how the developmental trajectory of these circuits may lead to the onset of anxiety during adolescence and, moreover, how effective pharmacological and psychological treatments may serve to shift the balance of activity within this circuitry to ameliorate negative bias symptoms. Together, these findings may bring us closer to a mechanistic, neurobiological understanding of anxiety disorders and their treatment.

## Introduction

Anxiety disorders are the most common cause of mental illness in the developed world, with large social, economic and psychological impacts (DiLuca and Olesen, 2014; Shin and Liberzon, 2009; Vos et al., 2016). A propensity towards the development of anxiety disorders is heritable (Hettema et al., 2005), often begins in childhood or adolescence (Beesdo et al., 2009; Pine, 2007) and persists into adulthood (Copeland et al., 2014; Craske et al., 2017). It is estimated that close to one in four people will suffer from an anxiety disorder – including generalised anxiety (GAD), post-traumatic stress disorder (PTSD), social anxiety or phobias – in their lifetime (Kessler et al., 2005, 2012), but currently available psychological and pharmacological treatments are effective for less than half of these individuals (Roy-Byrne, 2015; Community and Mental Health team, 2014) and progress in the discovery of anxiolytic drugs has been slow (Griebel and Holmes, 2013). One reason for this treatment gap is that we have a limited understanding of the biological mechanisms by which anxiety symptoms emerge or how these mechanisms are modulated by our current interventions. As such, we struggle to develop new treatments that can modulate known biological targets. Moreover, it is increasingly clear that our current diagnoses, based largely on self-reported symptoms, do not map clearly onto underlying biology or indeed onto the latent structure of the self-reported symptoms themselves (Cuthbert and Insel, 2013; Kotov et al., 2017). Indeed, factor analyses suggest that many categorical disorders consist of shared symptoms which are similar manifestations of relatively few underlying dimensions (Caspi et al., 2014; Clark et al., 2017; Kaczkurkin et al., 2017; Kotov et al., 2017; Krueger and Eaton, 2015; Lahey et al., 2012, 2017).

Recent efforts such as the Research Domain Criteria (RDoC; Insel et al., 2010), therefore, attempt to re-frame the investigation of psychiatric disorders by advocating a trans-diagnostic approach focusing on the neurobiological mechanisms underpinning symptoms that cut across traditional categorical diagnoses. In particular, one domain within the RDoC, *Negative Valence Systems*, includes responses to aversive situations such as fear, anxiety, sustained threat and loss (/reward omission) that overlap with a key concept from the clinical psychology literature – *negative affective bias*. Negative biases in cognition are thought to promote and uphold key symptoms of many psychiatric conditions but are especially prominent in anxiety disorders, perpetuated by anticipation of – and uncertainty about – future events (Grupe and Nitschke, 2011, 2013). Although none of our current treatments or diagnoses are based on a mechanistic neurobiological understanding of negative bias, recent work has begun to delineate the role that interactions between the prefrontal cortex (PFC) and subcortical regions such as the amygdala play in the manifestation of negative bias in anxiety. It is this circuitry that is the focus of the present review.

## Negative bias in anxiety

Anxiety disorders are characterised by a general ‘negative bias’ in both attention and memory towards affectively negative (/threatening/aversive) information that promotes and upholds the anxious state while having knock-on effects in a wide range of other cognitive functions (e.g. learning, inhibitory control; Craske et al., 2017; Robinson et al., 2013a). It has been widely shown, for instance, that people with anxiety tend to interpret neutral information in a more negative light, have maladaptive attention biases towards threat even when threats are not immediately present or relevant (this may be particularly prominent in some subtypes of anxiety such as social anxiety disorder; Abend et al., 2017) and have a bias towards learning about negative information (Abend et al., 2017; Hakamata et al., 2010; Monk et al., 2006; Okon-Singer and Aue, 2017; Roy et al., 2008). It is of course possible to break negative biases into specific subtypes of bias (for a comprehensive attempt to do this, see Grupe and Nitschke, 2013), but here we aim to build a broad preliminary model across disparate animal and human experimental data, along with clinical data, with the goal of drawing holistic conclusions. Similarly, the term *negative bias* of course encompasses myriad constructs, including a distinction made by many researchers between fear and anxiety (Davis et al., 2010), as well as subcategories of learned versus prepotent fears (LeDoux, 2000; Phelps et al., 2004). However, in this review, we broadly focus across the *Negative Valence Systems* domain of the RDoC (Insel et al., 2010) in an attempt to identify common patterns and build a simple model of how negative bias is generated at the neurobiological level. If we were to be too fine-grained in our definitions of negative bias, we will find ourselves with multiple non-overlapping studies, which would limit interpretation.

To demonstrate our aim of bridging disparate experimental literature and clinical utility, consider Beck’s (1967) early theory of elevated negative bias (or ‘*negative schemata*’). In this framework, Beck proposed a cognitive triad illustrating a cycle among a negative view of the world, the self and the future. This model encompasses wide-ranging cognitive functions from memory to attention but nonetheless forms the basis of successful psychological treatments such as cognitive-behavioural therapy (CBT), highlighting potential advantages in taking a broad approach to linking cognitive research with clinical practice.

We therefore review converging evidence across humans and animal models suggesting that negative bias may arise, at least in part, from activity within prefrontal regions and their interactions with subcortical regions (Etkin and Wager, 2007; Shin and Liberzon, 2009). These circuits may underpin the ability to engage or disengage attention from threats and may be critical to understanding the mechanistic basis of negative bias as well as its development and treatment (Aue and Okon-Singer, 2015; Cisler and Koster, 2010; Dodd et al., 2017).

## Animal models of negative bias

Research falling within the *Negative Valence Systems* domain of RDoC in animal models often makes a distinction between fear and anxiety. In the psychological literature, anxiety is defined as a prolonged state of heightened anticipatory arousal, often prompted by distal or unpredictable threats (Davis et al., 2010). Fear, on the contrary, is conceptualised as a ‘fight or flight’ reaction and typically involves active defence against immediate threat, usually dissipating upon removal of the threat. In ‘real-world’ terms, a person’s reaction to a spider on the table in front of them might elicit a fear response (which may be exacerbated in cases of phobia), whereas the knowledge that a spider might be in the room but uncertainty of its location might elicit anxiety. Elevations in both responses could of course play a key role in driving negative affective bias (although see Fox and Shackman, 2017; Shackman and Fox, 2016, for suggestions of why this explicit distinction between fear and anxiety might be problematic with regard to the underlying neurobiology).

Animal models are particularly useful in the investigation of the neural basis of anxiety and fear because of their cross-species overlap in the neural circuitry underlying these processes (Davis, 2000; LeDoux, 2000); cross-species functional homologues of brain circuitry can inform translational research across humans and rodents and can provide testable models, even if the circuits themselves are not directly conserved across species. For instance, startle response, in which whole-body jump is typically used as an index in rodents, is paralleled by an eye-blink response in humans (Davis, 2001) and is a reliable experimental measure of aversive responding on some cognitive tasks (Aylward and Robinson, 2017) but not necessarily on others (Bradford et al., 2015). This startle reflex is heightened by both fear and anxiety states across rats and humans (Grillon and Davis, 2007; Grillon et al., 1991), but the subcortical circuitry responsible may differ (Robinson et al., 2012a). Specifically, fear responses are associated with the central amygdala, anxiety responses are associated with the bed nucleus of the stria terminalis, and both fear and anxiety responses are associated with the basolateral amygdala (BLA; Davis et al., 2010; Sengupta et al., 2017; Tovote et al., 2015), although this distinction has been challenged and warrants further investigation (Gungor and Paré, 2016; Shackman and Fox, 2016). Nevertheless, taken together, rodent and human work points to the clear role of subcortical regions, and the extended amygdala in particular (along with its inter-connections and external projections), in driving aversive/fear responding and hence negative bias (Boeke et al., 2017; Campese et al., 2015, 2017; Sengupta et al., 2017; Terburg et al., 2012; Tovote et al., 2015).

However, regions of the brain rarely, if ever, work in isolation. Within the hierarchy of neural processing, these subcortical regions also interact with ‘higher’ cortical areas. This is perhaps best illustrated by work exploring the weakening of learned aversive responses during *extinction*. During fear extinction, a cue which previously indicated the onset of an aversive event no longer predicts a negative outcome, so the individual must ‘extinguish’ their original aversive response. Animal models of conditioned fear extinction indeed implicate subcortical regions such as the BLA, but they extend the circuitry to encompass medial prefrontal cortical regions as well. In particular, within the rodent PFC, subdivisions of the infralimbic (IL) and the prelimbic (PL) have been posited to play distinct roles in the expression and extinction of conditioned fear, with the IL supporting fear extinction as expressed by the amygdala and the PL conversely promoting fear expression as expressed by the amygdala (Klavir et al., 2017; Morgan and LeDoux, 1995; Sierra-Mercado et al., 2011; Vidal-Gonzalez et al., 2006). In monkeys, activity in the dorsal anterior cingulate (dACC) is correlated with the BLA during fear learning and memory acquisition (Klavir et al., 2013; Livneh and Paz, 2012), and in rodents, PL response to cues predicting an aversive event increases post-fear learning (Burgos-Robles et al., 2009). Using pharmacological or electrical stimulation and optogenetic approaches, it has been shown that, on the other hand, increased activity in the rodent IL predicts fear extinction in the amygdala (Do-Monte et al., 2015; Klavir et al., 2017; Milad and Quirk, 2002, 2012). Having said that, recent rodent work has suggested that this dissociation may not be as clear-cut as previously thought. For example, the role of the IL in fear extinction has been challenged in optogenetics work showing that extinction recall was intact after the silencing of IL neurons and that stimulation of ventromedial prefrontal cortex (vmPFC) inputs to the amygdala facilitated extinction memory formation but not retrieval (Bukalo et al., 2015; Do-Monte et al., 2015). Moreover, the distinction between PL/dorsal and IL/ventral prefrontal regions being responsible for fear expression and suppression, respectively, has been challenged (Giustino and Maren, 2015). For instance, rodent work has shown that these regions have structurally similar projections to the amygdala (Cho et al., 2013; Gutman et al., 2012; Hübner et al., 2014; Pinard et al., 2012), and functionally dichotomous distinctions between these regions have been shown in the opposite direction than was initially postulated (Chang et al., 2010).

This rodent research nevertheless highlights a key potential mechanism of negative bias; namely that the overall expression of aversive responding may be held in the balance of opposing circuitry. Thus, whether negative bias is expressed or dampened may depend on whether one of these circuits is able to override the other, with sub-regions of the medial prefrontal cortex (mPFC) playing a key role in arbitrating this response. Indeed, across a range of paradigms in rodents, the PFC has been shown to play a regulatory role over BLA activation during fear expression, social interaction and anxiety-related behaviours (Bickart et al., 2014; Bremner, 2004; Davis, 1998; Felix-Ortiz et al., 2013; Janak and Tye, 2015; Kling and Steklis, 1976).

That said, the implicit implication that the PFC is a ‘top-down’ regulator of the amygdala during fear extinction should be challenged. Optogenetics research in rodents has shown *bi-directional* effects of modulating BLA projections to the IL and PL during a number of behavioural assays assessing anxiety-like behaviour (Courtin et al., 2014; Felix-Ortiz et al., 2016; Herry et al., 2008; Laviolette et al., 2005). Specifically, BLA activity projecting *up* to PL regions is increased during fear conditioning (Senn et al., 2014), while fear extinction also enhances activity in BLA projections *up* to the IL (Milad and Quirk, 2002; Senn et al., 2014). Moreover, recordings from non-human primates further support this bi-directional effect during fear learning (Klavir et al., 2013). In other words, it is not so much that the PFC ‘regulates’ the amygdala but rather the reciprocal relationship of information flow between these regions in a circuit that drives the overall output.

Collectively, animal work therefore suggests a putative neural mechanism of negative bias; one (bi-directional PL–amygdala) circuit may serve to facilitate negative bias, while another (bi-directional IL–amygdala) circuit may serve to suppress negative bias (Calhoon and Tye, 2015). This simplified heuristic provides a framework with which to consider neurobiological research in anxious humans.

## Neurobiological basis of negative bias in humans

Perhaps unusually for a symptom related to psychiatric disorder, negative bias in anxiety can be adaptive. For example, when one is walking home late at night and hears an unexpected noise down a dark alley, an appraisal of this situation as potentially threatening raises awareness, preparing the body’s fight or flight response in the event of immediate danger. In other words, negative bias in anxiety can promote harm avoidance. However, if this heightened anxiety and negative bias does not subside when one is subsequently safe at home, this response becomes maladaptive and can impair daily functioning (i.e. it transitions into a pathological state). Thus, it was suggested by the pioneers of CBT that a biased appraisal of threat (i.e. negative bias) leading to catastrophising or excessive worry is a central characteristic of anxiety disorders (Beck and Clark, 1997). Dorsal regions of the PFC (dorsomedial PFC (dmPFC) and dACC) seem to be associated with this behavioural response at the neural level; these regions are activated during conscious threat appraisal in healthy individuals and have been shown to be overactive during threat appraisal in pathological anxiety (for review, see Kalisch and Gerlicher, 2014).

Consistent with the animal work highlighted above, pathological negative bias in humans may in fact result in part from an inability to extinguish conditioned fear responses driven in turn by this altered, worry-related PFC responding (Milad et al., 2007; Rothbaum and Davis, 2003). Healthy subjects show increased activation in the vmPFC during acquisition and retrieval of extinction (Kalisch et al., 2006; Milad and Rauch, 2007; Phelps et al., 2004), which has led to the suggestion that this region in the human brain might be functionally (albeit perhaps not structurally) homologous to the rodent IL. On the other hand, patients with anxiety disorders have shown reduced activation in the vmPFC along with increased activation in the dACC, leading to the suggestion that the dACC might be functionally homologous to the rodent PL (Milad et al., 2009).

Consistent with this proposition, at the neural level, the dACC and adjacent dmPFC have been implicated in the appraisal and expression of fear (Etkin et al., 2011; Vogt, 2005), as well as the anticipation of emotional stimuli (Erk et al., 2006). Moreover, Kalisch and Gherlicher (2014) argue that the dACC/dmPFC can be further subdivided into an anterior part, the rostral dACC/dmPFC and a posterior part, with the rostral but not posterior part implicated in conscious threat appraisal and worry (Kalisch and Gerlicher, 2014; Mechias et al., 2010). Thus, activity in *dorsal* PFC regions is broadly associated with *increased* negative bias. The dACC has also been linked to the adaptive control of behaviour as well as the risk of development of anxiety disorders (Cavanagh et al., 2017; Goodkind et al., 2015; McTeague et al., 2017; Meyer, 2017).

Regarding ventral regions, studies in healthy adults (Bishop, 2007) as well as adults (Etkin and Wager, 2007; Milad et al., 2007; Price and Drevets, 2012) and children and adolescents (Guyer et al., 2008; Monk et al., 2006; Strawn et al., 2012) with anxiety disorders have shown abnormal function in orbitofrontal cortex (OFC) and ventrolateral PFC (VLPFC). The cause and effect of such abnormalities have been studied in non-human primates through lesions to the anterior OFC and VLPFC (Agustín-Pavón et al., 2012; Izquierdo and Murray, 2005; Kalin et al., 2007; Machado and Bachevalier, 2008), with findings largely showing increased anxiety during fear conditioning paradigms when these regions are lesioned. Thus, broadly speaking, activity in *ventral* cortical regions is associated with *reduced* negative bias (although it should be noted that this association may not be as consistent as previously thought (cf. Shackman et al., 2011).

The hippocampus is another structure that has been hypothesised to play a critical role in the pathophysiology of anxiety. Specifically, this region is a key mediator of the acquisition and expression of learned fear, as demonstrated by a number of early lesion studies showing that hippocampal lesions dampened fear response to previous learned associations (Kim and Fanselow, 1992; Phillips and LeDoux, 1992; Selden et al., 1991). Studies in both human and rodents suggest that this region integrates contextual information during fear conditioning and may regulate context-dependent recall after extinction (Giustino and Maren, 2015). The rodent PL and IL receive excitatory inputs from both the dorsal and ventral hippocampus (Little and Carter, 2013), and it has been suggested that, similar to the amygdala, these projections may inhibit downstream mPFC outputs (Sotres-Bayon et al., 2012). In humans, Linnman et al. (2011) demonstrated that fear (elicited by electric shock expectation) was associated with increased connectivity between the hippocampus and the vmPFC, and decreased connectivity between the hippocampus and the red nucleus midbrain region, suggesting that the hippocampus may facilitate a switch between what they term a ‘fear’ network and a ‘resting’ network.

However, as highlighted by the rodent literature above, negative bias is not so much driven by regions acting in isolation. Rather, it is the cortical–subcortical *circuitry* that is important for anxiety response. For example, Kalin et al. (2016) used a viral vector approach in primates to demonstrate a relationship between overexpression of corticotropin-releasing hormone (CRH) in the dorsal amygdala and increased defensive behaviour during exposure to threat. Moreover, this link between metabolism and behaviour has also been observed in rodents and was associated with functional connectivity between the dorsal amygdala and OFC (Regev et al., 2012). To this end, connectivity between the dACC/dmPFC and the amygdala has been implicated in the pathophysiology of anxiety in humans. Structurally, the integrity of white matter tracts between the amygdala and the PFC has been shown to predict individual differences in trait anxiety (Kim and Whalen, 2009). Functionally, Robinson et al. (2012a) studied the role of these regions in negative bias during induced anxiety in healthy individuals. They found that connectivity increased during the processing of threatening stimuli (fearful faces) selectively in the context of induced anxiety. Moreover, the strength of this connectivity was positively correlated with participants’ subjective ratings of anxiety, as well as the extent of negative bias in behavioural responding (as indexed by a threat-by-valence interaction in reaction times, driven by a valence-specific reduced reaction time to fearful faces under threat vs safe conditions), suggesting a key mediating role for dACC/dmPFC–amygdala circuitry in driving negative bias. Critically, coupling within this same circuitry was shown to be elevated *at baseline* in individuals with an anxiety disorder (Robinson et al., 2014; in the absence of induced anxiety), suggesting that the same circuitry which can be selectively engaged and disengaged in healthy controls is more persistently engaged in patients with clinical anxiety, thereby providing a route by which adaptive anxiety can transition into a maladaptive state. Across both studies, however, the correlation between the amygdala and dorsal cortical regions was *positive*. In other words, activity in the dorsal cortical regions increases as activity increases in the amygdala and vice versa. The role that this circuit seems to play in threat responding therefore appears somewhat analogous to the role of the PL in rodents. Thus, a human functional homologue of the rodent PL–amygdala circuit may drive increased threat responding and negative affective biases in anxiety disorders.

However, rodent work has also highlighted the contrasting role of the inhibitory IL circuit (Kim et al., 2011a). To this end, another study in humans (Vytal et al., 2014) expanded this putative circuitry to encompass a reciprocal inhibitory circuit. Specifically, inducing anxiety during an adapted resting-state scan replicated *positive* dmPFC–amygdala coupling, but at the same time enhanced *negative* coupling between a ventral medial prefrontal region and the amygdala. In other words, while increased dorsal activation was associated with increased amygdala activation, increased ventral activation was associated with *decreased* amygdala activity (Vytal et al., 2014). Earlier positron emission tomography (PET) studies in humans have shown similarly contrasting relationships between prefrontal and subcortical regions (Linnman et al., 2012a, 2012b). For example, during extinction training, resting amygdala metabolism positively predicted vmPFC activation and negatively predicted dACC activation, but during extinction recall, these relationships were in the opposite direction (Linnman et al., 2012a). In a rodent study investigating the impact of early-life environmental stress, Johnson et al. (2018) showed that stress was related to *increased* amygdala–PFC and amygdala–hippocampus coupling and that this connectivity was related to anxiety-like behaviours in a translational model of early-life stress. Thus, these studies demonstrate that the relationship between distinct mPFC regions may have opposing effects on aversive responding. Another study of resting-state functional connectivity in healthy humans showed that those individuals who reported high levels of anxiety were characterised by negatively correlated amygdala–vmPFC connectivity, while this connectivity was positively correlated in those reporting low levels of anxiety (Kim et al., 2011b). Moreover, amygdala–dmPFC connectivity was negatively correlated only in those reporting low anxiety. More dorsal regions of the PFC (like the PL in rodents) may increase aversive responding, while more ventral regions (like the IL in rodents) may reduce aversive responding. Moreover, the nature of these functional imaging connectivity analyses means that they are non-directional. In other words, it is not possible to say whether one region is driving the other – it is simply a correlation. Given the bi-directional nature of the rodent work highlighted above, these circuits therefore should not be considered ‘top-down’ or ‘bottom-up’; rather, the overall reciprocal cortical–subcortical interaction likely drives the ultimate behavioural expression.

Together, these findings highlight the value of translational research. A model of cortical–subcortical interactions during negative bias inspired by rodent work provides a framework within which to consider the role of neural circuitry in negative bias in humans.

## Development

The work reviewed above therefore suggests that medial prefrontal–amygdala interactions may drive the negative bias symptoms that are a core feature of anxiety disorders. However, how these mechanisms develop and persist across the lifespan remains unclear. If we want to target these symptoms and intervene early, it is important to determine when and how alterations to these circuits emerge.

Within the general population, pathological anxiety commonly emerges during childhood or adolescence and reflects a combination of genetic factors and early-life experiences (Pine, 2007). ‘Anxious temperament’ is considered to be a stable trait across time, and those with extreme levels of such traits are at a higher risk for developing clinical or pathological anxiety (Arnaudova et al., 2013; Jones, 2013; Nugent et al., 2011). Similarly, the stable traits of ‘behavioural inhibition’, a temperament characterised by a tendency to withdraw from new situations (Kagan et al., 1987; Svihra and Katzman, 2004) and, more broadly, ‘dispositional negativity’ (Shackman et al., 2016) are thought to be early phenotypes of anxiety disorders. There is evidence that anxiety-related amygdala abnormalities and affected top-down prefrontal regulation originate early in development (Clauss and Blackford, 2012; Kalin, 2017). Moreover, it has been estimated that 50% of children showing increased behavioural inhibition in childhood will later develop stress-related psychopathology (Clauss and Blackford, 2012). This is paralleled by findings of reduced amygdala–dorsolateral prefrontal cortex (dlPFC) coupling in preadolescent children diagnosed with an anxiety disorder as well as in young non-human primates with elevated levels of traits related to anxious temperament (including heightened behavioural inhibition; Birn et al., 2014). Although more longitudinal studies are needed to confirm this, this evidence suggests that rapid changes in the mPFC and the later maturation of amygdala–cortical connections during adolescence, a period recently suggested to encompass 10–24 years of age (Sawyer et al., 2018), may contribute to the emergence of anxiety during a specific developmental window (Andersen, 2003; Casey et al., 2008). Indeed, prospective studies in humans (Giedd et al., 1999; Jones et al., 2017; Kalin, 2017; Swartz and Monk, 2013) as well as rodent studies (Arruda-Carvalho et al., 2017; Cohen et al., 2013; Gee et al., 2013a; Pattwell et al., 2016) have shown that this period constitutes a window of heightened risk for the development of anxiety. However, vast structural brain changes have also been observed during childhood, suggesting that children are subject to a heightened vulnerability to environmental impacts which may influence the development of anxiety even before the onset of adolescence. Indeed, behaviourally inhibited temperament has been observed in young children who later develop anxiety, with similar neural circuitry alterations linking these phenotypes (Buzzell et al., 2017; Gold et al., 2016; Sylvester et al., 2016).

One influential idea common to the human and rodent developmental literature is that learned fear associations (i.e. memories) from early life are important contributing factors to the subsequent development of anxiety disorders (Britton et al., 2011; Glenn et al., 2012; Jacobs and Nadel, 1985). Cross-species animal work (Harlow and Harlow, 1965; Hess et al., 1962) has shown that fear learning is characterised by approach behaviour (such as maternal attachment or odour approach) in infants, but is characterised by almost diametrically opposed avoidance behaviour (such as maternal or odour avoidance or avoidance of elevated/open areas in typical rodent paradigms) in adults (for an extensive review, see Ganella and Kim, 2014). This also suggests that at some point during development, there is a change in the underlying neurobiology promoting this behaviour.

Integrating this within the circuitry framework of the present review, Chan et al. (2011) inactivated PL in juvenile, preadolescent and adult rats and found that PL inactivation significantly *reduced* freezing behaviour, as would be predicted by the above reviewed evidence, but that it only did so in adolescent and adult rats, suggesting that the role of different medial prefrontal regions in negative bias changes across development. In other words, differences in fear responding, mediated by amygdala–medial prefrontal pathways, may partially be a result of a more protracted course of development and reorganisation in these cortical–subcortical pathways (Arruda-Carvalho et al., 2017; Ganella and Kim, 2014; LeDoux, 2000; Pattwell et al., 2016). Similar developmental changes in prefrontal–subcortical negative bias circuitry are also seen in humans; in typically developing humans, mPFC–amygdala connections are immature during childhood and strengthen to adult levels during adolescence (Gee et al., 2013a, 2013b), and structural changes in white matter have been shown to mediate amygdala function in adolescents (Swartz et al., 2014). Moreover, early perturbations in medial prefrontal circuitry have been implicated in the development of anxiety and depression. For example, a preliminary study in adolescents with depression found that patients had decreased functional connectivity in a subgenual (ventral) anterior cingulate (ACC)-based network compared to healthy adolescents (Cullen et al., 2009). Moreover, negative coupling within vmPFC–amygdala circuitry during fear extinction was recently shown only in adults and not adolescents (Ganella et al., 2017). These ventral regions may reflect overlapping human homologues of the rodent IL. Thus, a developmental delay in the ability to engage the circuitry that can *dampen* negative bias might explain the emergence of anxiety disorders during adolescence. This work is in its infancy, but the concept of reciprocal cortical–subcortical circuits again provides a framework with which to consider the emergence of anxiety and negative bias during development.

## Treatment

If prefrontal–subcortical circuitry is critical in the development and manifestation of negative bias, then modulation of this circuitry should serve to modify negative affective biases and hence treat symptoms. The first-line treatments for clinical anxiety are serotonergic medication and psychological therapy. Emerging evidence suggests that successful response to both types of treatment may also depend on this prefrontal–subcortical circuitry.

### The role of serotonin in pharmacological treatment

Serotonin (5-hydroxytryptamine (5-HT)) has long been implicated in the neuropsychopharmacology of anxiety (Dayan and Huys, 2009; Harmer et al., 2009, 2011), largely because selective serotonin reuptake inhibitors (SSRIs) are the most common and effective pharmacological treatment for anxiety disorders (Harmer et al., 2009, 2011). It is thought that serotonin plays a particular role in maintaining the balance between the processing of appetitive and aversive information (Cools et al., 2008; Crockett et al., 2009; Robinson et al., 2012b) and more precisely in the inhibition of PFC-linked neural circuitry important for driving negative bias (Crockett et al., 2009; Robinson et al., 2013b).

The impact of serotonin in healthy humans can be studied by acute tryptophan depletion – a dietary manipulation that temporarily reduces serotonin levels (Crockett et al., 2012). Reduced serotonin has been shown to *increase* positive coupling within the same circuit shown to be elevated by induced anxiety (Vytal et al., 2014) and at baseline in individuals with an anxiety disorder (Robinson et al., 2014), suggesting that serotonergic drugs (which putatively elevate serotonin availability) may work by *reducing* activity within this dorsal prefrontal circuit (Robinson et al., 2013b), thus reducing negative bias. By contrast, a study using a different paradigm showed that tryptophan depletion can also *decrease* coupling between the amygdala and a more ventral prefrontal region (Passamonti et al., 2012). Moreover, direct reductions in ventrally located orbitofrontal serotonin in the marmoset can *increase* negative bias (Rygula et al., 2015). Within the framework described above, this suggests that serotonin can also serve to *promote* ventral PFC circuits that inhibit aversive processing while *inhibiting* dorsal PFC circuits that promote aversive responding. As such, pharmacological treatments may work by restoring the balance between the circuits that, respectively, promote and inhibit negative bias. Recent work also suggests that the influence of serotonergic drugs on this circuitry might be mediated by genetic factors (Perna et al., 2005; Santangelo et al., 2016), which may in turn explain why such medications only work for a subset of anxious patients.

### Psychological treatment

CBT is the most common psychological intervention used to treat anxiety and is based on the premise that negative biases in thoughts and actions can be shifted through cognitive reappraisal and emotion regulation strategies (Beck and Clark, 1997). There have been numerous studies (see review by Brooks and Stein, 2015) which suggest that CBT modulates prefrontal–subcortical interactions. Indeed, baseline medial prefrontal and amygdala activity might even predict treatment response to CBT in anxiety (Klumpp et al., 2017). For instance, Shou et al. (2017) showed that functional connectivity between the amygdala and the fronto-parietal network increased in patients with major depressive disorder (MDD) or PTSD who underwent a course of CBT compared to controls, supporting a mechanism by which this circuitry may interact with psychological intervention (although it should be noted that this study did not include a patient group that did not undergo CBT, so the specificity of these results is difficult to quantify). Similarly, a study of adolescents with anxiety assessed whether CBT combined with attention bias modification therapy (ABMT) was more clinically effective than CBT alone and whether this treatment response could be predicted through pre-treatment amygdala-based functional connectivity (White et al., 2017). This study found that patients differed from controls in amygdala–insula connectivity on a threat attention task. Moreover, while both CBT groups showed clinical improvement, the combined CBT + ABMT group showed the greatest reduction in symptoms and that baseline amygdala functional connectivity differentially predicted the level of treatment response in patients.

However, whether these changes in cortical–subcortical circuits are driven by CBT, or whether they simply reflect reduced overall anxiety and negative bias per se, is unclear. To this end, basic research has attempted to determine causality. Specifically, it has been shown that in healthy individuals, simple attentional instruction can alter the engagement of affective-bias-related dmPFC–amygdala circuitry (Robinson et al., 2016). When subjects are instructed to pay attention to neutral aspects of compound cues (rather than the affectively salient components of the same cues), anxiety-induced amygdala–dmPFC coupling (as seen above; Robinson et al., 2012a) is down-regulated. This suggests that psychological treatments such as CBT may reduce negative bias by down-regulating the dorsal PFC–amygdala circuitry that promotes negative bias. In the context of threat processing, there has been limited work showing whole-brain increased ventrolateral prefrontal activation in anxious youth who underwent CBT relative to controls (Maslowsky et al., 2010), as well as reduced dorsomedial prefrontal activation post-CBT relative to pre-CBT in individuals with social anxiety (Klumpp et al., 2013). Nevertheless, the role that CBT plays in dorsal versus ventral prefrontal–amygdala circuitry in humans has not been systematically studied. Moreover, recovery rates of patients with anxiety undergoing psychological treatment are less than 50% (Community and Mental Health team, 2014), so it is plausible that these mechanisms are again only relevant in a subset of patients.

## Conclusions and future directions

**Figure 1.**
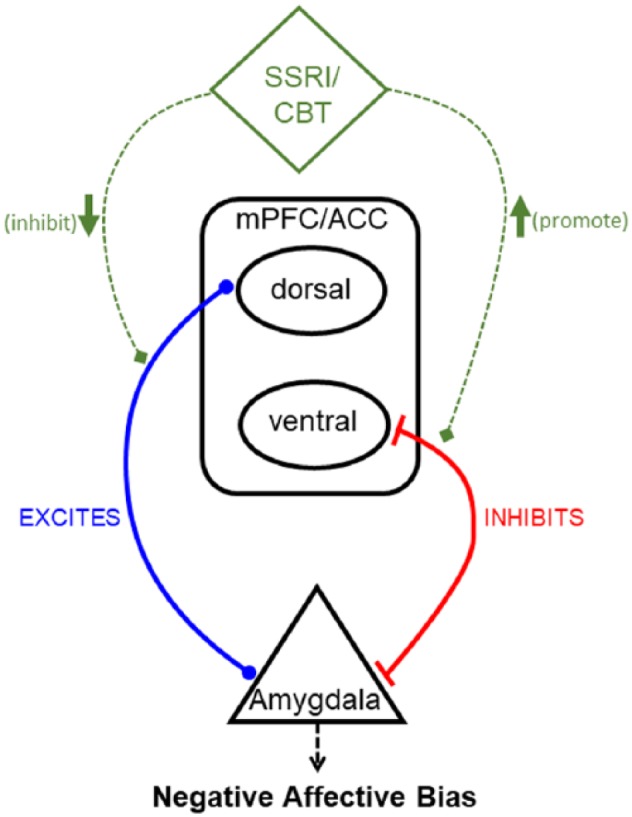
Schematic summarising findings and proposed simplified model of negative affective bias in anxiety. Bi-directional excitatory connections between dorsal regions of the mPFC/ACC and the amygdala promote negative bias, while inhibitory connections between ventral regions coupled with the amygdala inhibit negative bias. The ventral inhibitory circuit may only fully develop in adulthood, meaning that adolescence is a period of high vulnerability to negative bias. Successful treatments (SSRIs and CBT) may be effective via promotion of the ventral circuit and inhibition of the dorsal circuit. ACC: anterior cingulate cortex; mPFC: medial prefrontal cortex; CBT: cognitive-behavioural therapy; SSRI: selective serotonin reuptake inhibitor.

In this review, we have outlined evidence across animals and humans suggesting that bi-directional prefrontal–subcortical circuits and their interactions may drive elevated aversive processing, or negative bias, in anxiety. Specifically, we point to converging evidence within the *Negative Valance Systems* domain of the RDoC which suggests that ventral PFC–subcortical circuitry in humans may be associated with reduced negative bias, while more dorsal PFC–subcortical circuitry may be associated with increased negative bias. Moreover, we provide evidence suggesting that the emergence of anxiety in adolescence may be a result of differential developmental trajectories of these circuits and that both pharmacological and psychological interventions might be effective by modulating the overall balance of these circuits in driving negative affective bias. These findings are summarised in Figure 1.

Nevertheless, it is still unclear exactly how we bridge the gap between brain and behaviour. Although we can associate these circuits with negative bias, we cannot yet say how exactly the underlying neuronal activity is translated into observable behaviour. One particularly promising avenue in this regard is the nascent field of computational psychiatry, which attempts to bridge the gap between brain activation and observable symptoms (Huys et al., 2016). Specifically, using mathematical theories of cognition and human behaviour, computational psychiatry aims to objectively quantify the calculations generated by neurons which shape behaviour (Huys et al., 2016). This work is in its infancy but has begun to delineate the computational basis of common symptoms in anxiety linked to negative bias, such as avoidance (Mkrtchian et al., 2017), risk aversion (Charpentier et al., 2017) and goal-directed behaviour (Carlisi et al., 2017; Gillan et al., 2014).

Furthermore, if we are to understand current findings in a truly generalisable context, it is critical to investigate these mechanisms in large-scale populations. Cohort studies are an ideal way to examine these questions at the population level, often sampling from a diverse community of individuals. Data sharing efforts have attempted to address this. For example, the ENIGMA consortium is an international collaboration of research centres which aims to combine neuroimaging and genetic datasets from sites around the world in an attempt to amass sample sizes large enough to detect very small effects in brain imaging and genetic data (Thompson et al., 2014). Moreover, the UK Biobank (Sudlow et al., 2015) is a consortium across 22 research centres in the United Kingdom with genetic and longitudinal physical health and behavioural data on over 500,000 participants, all of which has been made open access. These are early efforts, particularly in the field of anxiety disorders, but promising mega- and meta-analyses have already come out of such efforts in other fields of psychiatry such as obsessive–compulsive disorder and schizophrenia (Boedhoe et al., 2016; De Wit et al., 2014; Van Erp et al., 2016).

Finally, it is important to investigate how these effects change over time. That is, are these mechanisms stable, or do they change across development to influence symptom onset and persistence? Longitudinal studies are critical for understanding these questions. There have been longitudinal studies investigating brain changes over time in adolescents (e.g. the IMAGEN study; Schumann et al., 2010), but this investigation needs to be scaled up to larger populations and multiple time points and age ranges if we are to truly understand the developmental changes that occur across the life course of anxiety disorders. One promising example of this work currently underway is the Adolescent Brain Cognitive Development study (ABCD; https://abcdstudy.org/index.html), which is the largest long-term longitudinal study of brain development in the United States, currently in the process of collecting biological and behavioural data on over 10,000 children aged 9–10. Similarly, to gain an understanding of the underlying genetic contributions of anxiety, it is important to investigate the extent to which certain features and symptoms are heritable. This can be achieved through longitudinal twin studies (e.g. the Twins Early Development Study (TEDS; Oliver and Plomin, 2007) and the Tennessee Twin Study (Lahey et al., 2008)), but many of the existing studies do not focus on brain imaging due to limited time and resources and the high cost involved in neuroimaging research. Regardless, observational population-based studies are an important complimentary approach to the small-scale case–control designs more frequently implemented in neuroimaging research on anxiety.

In conclusion, work has begun to delineate overlapping neural networks involving the PFC and subcortical regions including the amygdala that may drive aversive responding and negative bias in both animals and humans. There is also promising evidence that pharmacological and psychological interventions can shape this circuitry and hence ameliorate negative affective bias. Future research should expand these findings to larger populations and investigate how these neural underpinnings arise in childhood/adolescence and change over time to shape behaviour.
